# Whole Genome Resequencing of Arkansas Progressor and Regressor Line Chickens to Identify SNPs Associated with Tumor Regression

**DOI:** 10.3390/genes9100512

**Published:** 2018-10-19

**Authors:** Bhuwan Khatri, Ashley M. Hayden, Nicholas B. Anthony, Byungwhi C. Kong

**Affiliations:** Department of Poultry Science, Center of Excellence for Poultry Science, University of Arkansas, Fayetteville, NC AR 72701, USA;. bkhatri@uark.edu (B.K.); amhayden@elanco.com (A.M.H.); nanthony@uark.edu (N.B.A.)

**Keywords:** tumor regression, *v-src*, SNPs, resequencing, chicken

## Abstract

Arkansas Regressor (AR) chickens, unlike Arkansas Progressor (AP) chickens, regress tumors induced by the *v-src* oncogene. To better understand the genetic factors responsible for this tumor regression property, whole genome resequencing was conducted using Illumina Hi-Seq 2 × 100 bp paired-end read method (San Diego, CA, USA) with AR (confirmed tumor regression property) and AP chickens. Sequence reads were aligned to the chicken reference genome (galgal5) and produced coverage of 11× and 14× in AR and AP, respectively. A total of 7.1 and 7.3 million single nucleotide polymorphisms (SNPs) were present in AR and AP genomes, respectively. Through a series of filtration processes, a total of 12,242 SNPs were identified in AR chickens that were associated with non-synonymous, frameshift, nonsense, no-start and no-stop mutations. Further filtering of SNPs based on read depth ≥ 10, SNP% ≥ 0.75, and non-synonymous mutations identified 63 reliable marker SNPs which were chosen for gene network analysis. The network analysis revealed that the candidate genes identified in AR chickens play roles in networks centered to ubiquitin C (*UBC*), phosphoinositide 3-kinases (*PI3K*), and nuclear factor kappa B (*NF-kB*) complexes suggesting that the tumor regression property in AR chickens might be associated with ubiquitylation, *PI3K*, and *NF-kB* signaling pathways. This study provides an insight into genetic factors that could be responsible for the tumor regression property.

## 1. Introduction

Chickens are not only an important supply of proteins for the human population but are also outstanding animal models in several fields of biology. They provide excellent opportunities for unraveling the genetic basis of phenotypic variations [1]. Their larger population size and increased longevity provide a greater chance for the evolution of their variants, that are then selected for important agronomic traits, providing an exceptional opportunity to discover novel functions of the specific genes [2,3]. There are diverse variants of chickens integrating various mutations that affect disease resistance and susceptibility, growth rate, body weight, muscle color, reproduction, behavior, feather color, structure and distribution, and comb shape [4,5,6,7,8,9].

Arkansas Regressor (AR) and Arkansas Progressor (AP) chickens are important animal models for studying the molecular basis of disease resistance and susceptibility. The AR and AP chickens were developed in 1965 by inbreeding of White Leghorn (susceptible to Rous Sarcoma virus: RSV) and Giant Jungle Fowl strains (resistant to RSV) [10]. The AR birds regress tumors induced by *v-src* oncogene of RSV, unlike the AP birds which develop malignant tumors upon *v-src* activation in connective tissue known as sarcoma. The tumor regression process in AR chickens may be due to the suppression of cell division, induction of apoptosis, DNA damage repair, or inhibition of metastasis by various tumor suppressor genes. In chicken models, tumor regression has been found to be strongly associated with both B complex haplotype, that encodes major histocompatibility complex (MHC), and non-MHC molecules which include T-lymphocytes and B-cell alloantigens [11,12,13].

Single nucleotide polymorphisms (SNPs) are very common genetic alterations and occur at a rate of ~5 SNPs per kilobase (kb) in chickens [14] and ~1 SNP per 1–2 kb in human [15]. An SNP in a coding region of DNA may change the encoded amino acid (nonsynonymous), thereby altering the structure and function of the encoded protein. Other SNPs may be silent (synonymous) in the coding region or simply occur in the noncoding region of chromosomal DNA. The SNPs may influence gene expression, messenger RNA (mRNA) stability, and localization of mRNAs/proteins in subcellular compartments; therefore, they may develop alteration of phenotypic traits [16]. They have become important biomarkers and are utilized for the study of population genetics and evolutionary changes [15,17].

Several genetic variation analyses are performed to identify SNPs associated with disease resistance traits in chickens [4,18,19,20]. However, there is limited information about the genetic factors responsible for disease resistance mechanisms against RSV in chickens. Therefore, this study was performed to identify SNPs responsible for resistance of RSV-induced tumor development in AR birds, unlike in AP birds. The SNPs resources presented here can be useful markers for understanding the disease resistance mechanisms in chickens.

## 2. Materials and Methods

### 2.1. Chicken Lines and DNA Preparation

Adult AP and AR chickens that are maintained by Dr. N. Anthony at the University of Arkansas (Fayetteville, AR, USA) were used for this study. A blood specimen (1 mL) was collected from 12 birds from each line following an animal use protocol approved by the University of Arkansas Institutional Animal Care and Use Committee (IACUC; approval number: 14012). Genomic DNA was extracted from whole blood samples using a QiaAmp DNA mini kit (Qiagen, Hilden, Germany) following manufacturer’s instructions. The quality of the DNA was checked using a NanoDrop 1000 spectrophotometer (Thermo Fisher Scientific Inc., Waltham, MA, USA) and agarose gel electrophoresis. Then, the 10 best quality samples from each line were pooled to represent each chicken line.

### 2.2. Illumina Sequencing and Sequence Assembly

Library construction and whole genome sequencing for the pooled DNA samples were performed by the National Center for Genome Resources (NCGR; Santa Fe, NM, USA). Illumina HiSeq 2 × 100 bp paired-end read method was used for genome sequencing. The quality of raw sequencing data was determined using FastQC toolkit [21] and low-quality reads were removed using reformat.sh in BBMap [22]. The clean reads were then aligned to the chicken reference genome sequence for Red Jungle Fowl (galgal5) retrieved from National Center for Biotechnology Information (NCBI). For the reference-based genome alignment, the NGen genome sequence assembly program of the Lasergene software package (DNAStar, Madison, WI, USA) was used. Assembly parameters were as follows: File format, Binary Alignment Map (BAM); mer Size, 21; mer skip query, 2; minimum match percentage, 93; maximum gap size, 6; minimum aligned length, 35; match score, 10; mismatch penalty, 20; gap penalty, 30; SNP calculation method, diploid Bayesian; minimum SNP percentage, 5; SNP confidence threshold, 10; minimum SNP count, 2; minimum base quality score, 5. After assembly, the SeqMan Pro program of the Lasergene package (DNAStar) was used for further analyses [23].

### 2.3. SNP Detection and Analysis

The JMP genomics (SAS Institute, Inc., Cary, NC, USA) program was used for filtering unique SNPs for tumor regression in the AR chickens. Single nucleotide polymorphism (SNPs) occurring in both AR and AP lines were removed, leaving behind the unique SNPs for each line. To identify highly fixed and homozygous SNPs, the SNPs were filtered based on SNP percentages (SNP%). The SNPs with a SNP% of ≥ 0.75 (for example, number of SNP = 3 of read depth = 4) were chosen. The 75% cutoff for SNP selection was set by considering potential sequencing errors that can be generated by the massively parallel sequencing method. Potential causal tumor regressing SNPs that induce non-synonymous changes in CDS (coding DNA sequences; protein coding) regions were chosen and unique SNPs in either AP or AR showing ≥ 10 read depths were selected as reliable SNPs. To reduce false positives, reliable SNPs chosen by criteria described above were confirmed by double-checking the initial assembly results with alignment view in SeqMan Pro program of Lasergene package (DNAStar).

### 2.4. Validation of SNPs

For validation purposes, nine different SNPs associated with the induction of amino acid changes in CDS regions were randomly selected and subjected to allele-specific polymerase chain reaction (PCR) using a greater number of birds. The intent of this assay is to validate the identified SNPs in the broader population. The validation assay of SNPs confirms: (1) That the nucleotide differences seen in the sequenced bird`s also occur in other birds of the same group; (2) that the sequence variants do in fact represent fixed differences. For this, 96 phenotypically verified birds each from AR and AP lines were used for blood sampling and then genomic DNA isolation. Genomic DNA was purified from whole blood using the Wizard SV 96 Genomic DNA Purification System (Promega; Madison, WI, USA) following the manufacturer’s instructions. The quality and quantity of isolated DNA were determined using a Nanodrop 1000 spectrophotometer (Thermo Fisher Scientific) and agarose gel electrophoresis. DNA from all samples were then diluted to 1 ng/µL in 96-well PCR formats. Allele-specific primers were designed corresponding to nine different SNPs based on the Red Jungle Fowl genome sequence (galgal5). Two sets of primers were designed with a common reverse primer for each SNP. The forward primer designated as F1 is the reference type whose terminal nucleotide at the 3’ end matches with a base in the reference genome. The forward primer designated as F3 is the SNP type and terminal nucleotide at the 3’ end matches with a base in the SNP. The third nucleotide from the 3’ end of both the forward primers (F1 and F3) were intentionally changed so that they mismatch with the genome in that position [24]. All primers were commercially synthesized by Integrated DNA Technology (Ames, IA, USA) (Table 1). Allele-specific PCR was conducted using F1 and F3 forward primers and a common reverse primer separately in 25 µL reaction volume in 96-well plates with cycle conditions as follows: 95 °C for 1 min, 35 cycles of amplification (95 °C for 30 s, 55 or 63 °C for 1 min, 72 °C for 1 min), and final extension 72 °C for 10 min in Applied Biosystems 2720 Thermal Cycler (Life Technologies, Carlsbad, CA, USA). Formation of allele-specific PCR products was determined by 1% agarose gel electrophoresis.

### 2.5. Ingenuity Pathways Analysis

Candidate genes (*n* = 58) retaining SNPs (*n* = 63) associated with the regression of tumors after the filtering process were analyzed using Ingenuity Pathway Analysis (IPA; Qiagen; www.ingenity.com) for understanding the gene ontology and molecular networks. Since IPA uses mechanistic pathways derived from human, mouse, and rat bioinformatics, functionalities for genes containing SNP in chicken were interpreted based primarily on mammalian biological mechanisms. The number of molecules in the network was set to the limit of 35, leaving only the most important ones based on the number of connections for each focus gene (a subset of uploaded significant genes having direct interactions with other genes in the database) to other significant genes [25].

## 3. Results and Discussion

### 3.1. Genome Sequencing and Assembly

**Figure 1:**
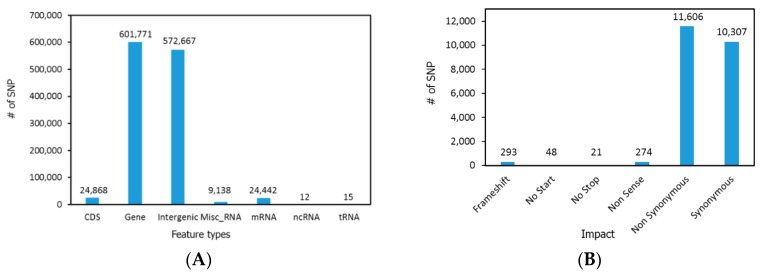
Distribution of single nucleotide polymorphisms (SNPs) in different regions of genome (**A**) and SNPs associated with different types of mutations (**B**). CDS: Coding DNA sequences; mRNA: Messenger RNA; ncRNA: Non-coding RNA; tRNA: Transfer RNA.

The results of the Illumina sequencing of pooled genomic DNA from 10 AR and AP chickens yielded approximately 55 and 69 million sequence reads, respectively, each 200 bp in length. Among these reads, approximately 80% were used for alignment while the remaining 20% were not aligned due to their lower sequence count scores. The sequencing coverage of AP and AR lines therefore reached 14× and 11×, respectively, to the Red Jungle Fowl genome, see Table 2. The 7.1 and 7.3 million SNPs examined in the AR and AP line, respectively, were found at the minimum of two read depths coverage. Most of the SNPs were found in large chromosomes 1 to 4 (data not shown). For identification of the signature genetic biomarkers that may be associated with the tumor regression trait in AR chicken lines, the unique SNPs that were present only in AR were selected by removing the SNPs that were also present in AP birds. Further steps of SNP filtration were carried out as described elsewhere by Jang et al. [23]. Briefly, the SNPs having SNP% ≥ 0.75, present in the CDS region only, associated with non-synonymous mutations (such as frameshift, nonsense, no-start and no-stop changes), and showing ≥ 10 read depths were considered as potential candidate SNPs and thereby included in this study. However, the process used in this study did not involve a typical SNP calling and filtering method based on quality score. From the unique AR SNPs, filtration based on SNP% ≥ 0.75 resulted in approximately 1.2 million SNPs identified throughout the AR chicken genome. Further grouping of SNPs based on feature type of chromosome regions showed that 24,868 SNPs were present in CDS region and about 50% were found in the intergenic region, see Figure 1A. Of these, non-synonymous mutations occupied ~50% of all SNPs found in CDS region, see Figure 1B.

The higher percentage of SNPs found in intergenic or regulatory regions in the AR chicken genome matched with a genome-wide association study (GWAS) study in chickens [26]. About 42% of AR SNPs in the protein coding region were associated with synonymous mutations that did not lead to amino acid changes, see Figure 1B. Though non-coding sequences and synonymous substitutions in protein coding regions are being considered as driver mutations leading phenotypic changes, in this study we aimed to identify the potentially causal SNP of non-synonymous mutations which were linked to changes in amino acid sequences followed by alterations of protein structures/functionalities that are responsible for the tumor regression property in AR chickens. A total of 12,242 SNPs were identified as linked with the induction of mutations such as non-synonymous, frameshift, nonsense, no-start and no-stop. This suggests that these SNPs may play roles in protein functions leading to the tumor regression property in AR chickens. Since the 10 genomic DNA samples per group were pooled for the genome resequencing reaction, SNPs showing ≥10 read depths (considered to be more reliable candidate genetic markers present in each coverage) were chosen for further analysis. It does not mean a particular SNP showing lower read counts is not important. When a number of genes retaining 12,242 SNPs were subjected to bioinformatic pathway analysis (IPA), it covered ~9000 genes, and almost all cellular and biochemical pathways were listed as functional mechanisms (data not shown). To specify certain functional pathways, we filtered SNPs with simple numerical read counts in each SNP position based on the consideration that the higher the counts, the more reliable the SNPs [6,23]. Using this approach, 63 SNPs remained, see Table 3. To reduce false positives due to possible errors in the assembly process, re-scanning of each SNP position for the 63 potentially more reliable SNPs was conducted using the Seqman-Pro viewer program (data not shown). These 63 candidate SNP markers were chosen for further bioinformatic pathway analyses. Remaining genes retaining SNPs will be investigated with an alternative approach, such as screening of a custom SNP array that contains 12,242 SNPs representing nonsynonymous amino acid changes.

### 3.2. SNP Validation

**Figure 2:**
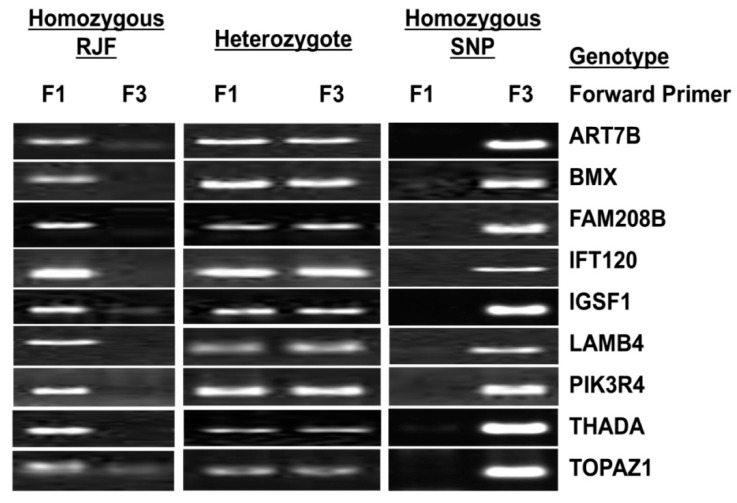
Nine candidate markers and different genotypes shown by allele-specific PCR in a larger population of AR and AP chicken lines. RJF: Red Jungle Fowl.

To verify the SNPs identified by genome resequencing, nine SNPs were randomly chosen from the 63 reliable candidate SNPs and subjected to allele-specific PCR in a greater number of birds, see Figure 2; specifically, 96 AR chickens with confirmed tumor regression property and 96 AP chickens with confirmed tumor progression property were used. The purpose of this validation is to confirm that identified SNPs present in the sequenced birds also occur in other birds of the same group to elucidate fixed differences between AR and AP groups. The results clearly showed the segregation of SNP genotypes as the majority of AR and AP birds showed SNP type and reference type, respectively, see Table 4. Thus, the 63 SNPs chosen in this study may become potential genetic biomarkers for tumor regression in AR chickens.

### 3.3. Ingenuity Pathway Analysis of Candidate Causal Genes

The IPA program was used to determine functional groups and networks analysis for genes containing amino acid changes in AR chickens. We used several online analytical tools (GO, KEGG, and Gorilla etc.), in addition to IPA, to analyze functions of the genes containing SNPs and obtained similar results of gene-to-gene interactions, function, and disease categories. Thus, the only bioinformatic pathway results generated by IPA will be discussed here in order to more clearly present the mechanistic results. The 63 SNPs were found in 58 genes associated with chromosomal open reading frames, and hypothetical proteins, see Table 5. The genes were further grouped into 89 functional groups (Supplementary Materials Table S1), that are directly or indirectly related to tumor development.

### 3.4. Gene Networks

Using IPA, molecular networks were generated using the interacting genes associated with amino acid changes in AR chickens based on functional knowledge inputs. A summary of the associated network functions of candidate genes is presented in Table 6.

**Figure 3:**
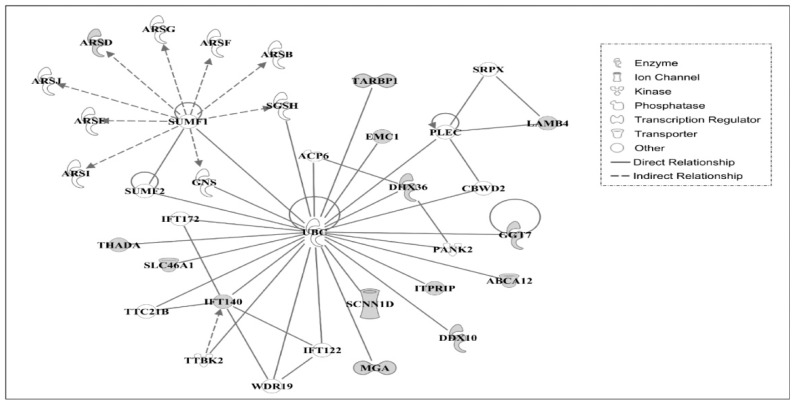
Gene network #1. Molecular interactions among the important focus molecules are displayed. Gray symbols show the genes found in the list of SNP while white symbols indicate neighboring genes that are functionally associated, but not included, in the gene list of SNP. Symbols for each molecule are presented according to molecular functions and type of interactions.

**Figure 4:**
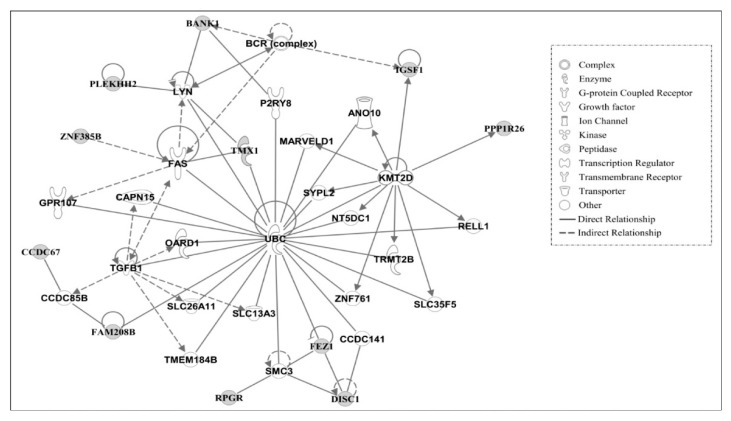
Gene network #2. Molecular interaction and symbols are the same as described in Figure 3.

The major functions of molecules associated with Network #1 are related to developmental, heredity, and metabolic disorders. Similarly, the top functions of molecules in Network #2 include cell death and survival, hematological system development and function, and humoral immune response, see Table 6. The molecules in Network #1, see Figure 3, and Network #2, see Figure 4, are centrally linked to ubiquitin C (UBC). The UBC functions in protein degradation, DNA repair, cell cycle regulation, kinase modification, endocytosis, and regulation of other cell signaling pathways. Ub ligase, an important enzyme in the ubiquitination process, which functions for ligating the substrate molecule to ubiquitin via lysine residue, may function in regulating the stability of oncogenes or tumor suppressors-proteins [27]. In AR chickens, the amino acid lysine was found to be changed to glutamic acid in FAM208B (family with sequence similarity 208, member B). Similarly, lysine residues in proteins LAMB4 (laminin, beta 4) and IFT140 (intraflagellar transport 140 homolog) were identified as having changed to arginine residues. This might suggest that various cellular processes involving protein degradation by altered ubiquitylation properties of proteins may play a significant role in the regression of tumors in AR chickens.

**Figure 5:**
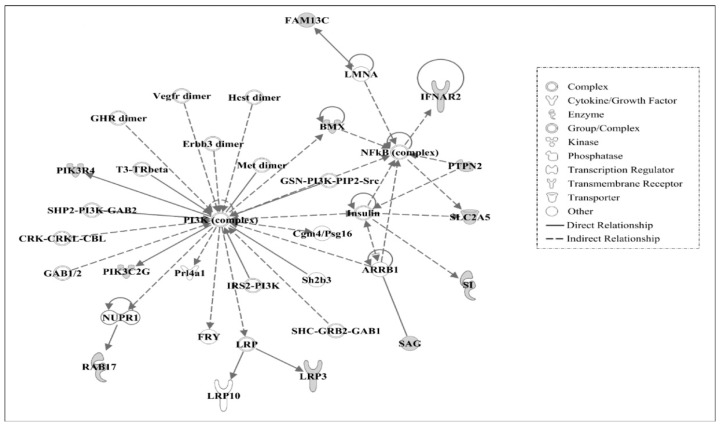
Gene network #3. Molecular interaction and symbols are the same as described in Figure 3.

The candidate genes in Network #3, see Figure 5, are associated with the signaling pathway of phosphoinositide 3-kinases (PI3K) and NF-kB (nuclear factor kappa-light-chain-enhancer of activated B cells) connected to Arrestin Beta 1 (ARRB1) with insulin signaling in the center. The top functions of the genes are related to developmental, gastrointestinal, and heredity disorders. It has been reported that the PI3K signaling pathway is crucial for several aspects of cell growth and survival. Recent human cancer genomic studies have shown that many components of this pathway are frequently targeted for the design of anticancer agents in humans by many aberrations including mutation, amplification, and rearrangement [28,29,30]. A study has also shown that the inhibition of the PI3K pathway leads to the partial inhibition of tumor growth [31]. In this study, the SNPs identified in the genes PIK3R4 and PIK3C2G, which are the components of PI3K pathway may have a role in the down-regulation of the PI3K pathway and may be responsible for the tumor regression trait in AR chickens. The NF-kB signaling pathway also plays a role in oncogenesis as it regulates the expression of genes involved in the development and progression of cancer such as proliferation, migration, and apoptosis [32,33]. It has been shown that blocking of PI3K leads to a marked reduction of constitutive NF-kB activity and promotes p53-mediated transcription. p53 is a crucial cellular protein that regulates the cell cycle and functions as a tumor suppressor, preventing oncogenesis [34,35]. Therefore, the down-regulation of both PI3K and NF-kB signaling pathways due to the SNPs present in components of the PI3K pathway might block the anti-apoptotic pathway and lead to apoptosis by p53 tumor-suppressing properties in AR chickens. BMX Non-Receptor Tyrosine Kinase (also known as ETK) which is present in Network #3 is a Tyrosine-protein kinase Tec family of kinase and is found to be expressed in endothelial lineages and some cancers such as breast and prostate. It has been shown to have anti-apoptotic properties in prostate cancer lines and regulate the PI3K signaling pathway [36,37,38]. Due to the occurrence of a single nucleotide polymorphism in the *BMX* gene, the oncogenic functions of the *BMX* gene may have been turned off along with its regulatory action on the PI3K signaling pathway. This may be another reason for the tumor regression character of AR chickens.

## 4. Conclusions

In this study, several candidate SNP markers which alter the amino acids residues associated with the tumor regression trait in AR chickens were detected through high-throughput genome re-sequencing. Based on bioinformatic studies, the reliable candidate genes containing SNPs were involved in ubiquitylation, PI3K, and NF-kB signaling pathways, suggesting their role in tumor regression in AR chickens. Future studies including screening custom SNP arrays that contain 12,242 SNPs representing nonsynonymous amino acid changes, allele-specific expression of the marker genes with candidate SNPs in target tissues, and more thorough bioinformatic pathway analyses will be performed to gain better insight into the mechanism of tumor regression in AR chickens.

## Figures and Tables

**Table 1:** Primers used for allele-specific polymerase chain reaction (PCR).

Gene	Primer Name	Oligo Sequence (5’ → 3’)	Annealing Temperature (°C)
*BMX*	BMX-F1	GAACTTACATACAGATCGTC	55
	BMX-F3	GAACTTACATACAGATCGTT	55
	BMX-R	CTTCCAACCCAAGCCATTAC	55
*FAM208B*	FAM208B-F1	CCACTCCTTGGTGGAGTATT	55
	FAM208B-F3	CCACTCCTTGGTGGAGTATC	55
	FAM208B-R	AGAAAGATGAGGATCGTGCG	55
*IFT140*	IFT140-F1	AAATCCATCAAGTTGATTAA	55
	IFT140-F3	AAATCCATCAAGTTGATTAG	55
	IFT140-R	TCTTTCTGAGAACGAAAGGG	55
*IGSF*	IGSF-F1	CAATGGGACTGTGCTGAGTC	63
	IGSF-F2	CAATGGGACTGTGCTGAGTT	63
	IGSF-R	TCTCAGGCAGAGGTGATGAT	63
*LAMB4*	LAMB4-F1	TCTCTTATTTGCGTTCAATT	55
	LAMB4-F3	TCTCTTATTTGCGTTCAATC	55
	LAMB4-R	TTGCAGATGAGAGTGTGCCT	55
*PIK3R4*	PIK3R4-F1	ACTAGGGTGAGATGTTTAAT	55
	PIK3R4-F3	ACTAGGGTGAGATGTTTAAC	55
	PIK3R4-R	GGGGATCATCAGAAGTCTGT	55
*THADA*	THADA-F1	ACAAACCATGCTGGCATACT	63
	THADA-F3	ACAAACCATGCTGGCATACC	63
	THADA-R	CAGGACATGCTAACCTCTGT	63
*TOPAZ1*	TOPAZ1-F1	AAGCTCTGGTAGGCTACGGG	63
	TOPAZ1-F3	AAGCTCTGGTAGGCTACGGT	63
	TOPAZ1-R	CAGGCCAGAATACTGCATCT	63

**Table 2:** Data from HiSeq and sequence assembly.

Line	# of Reads	# of Reads Aligned	# of Reads Not Aligned	Coverage	Total # of SNP
AP	69,221,284	55,224,050	10,903,306	14×	7,372,778
AR	55,368,344	44,328,649	8,551,173	11×	7,173,788

AP: Arkansas Progressor; AR: Arkansas Regressor.

**Table 3:** The 63 reliable marker SNPs that induced amino acid changes showing ≥ 10 read depths.

Contig ID	C	Ref Pos	Ref Base	Called Base	Impact	SNP%	Feature Name	DNA Change	AA Change	Depth	A Cnt	C Cnt	G Cnt	T Cnt	Del
NC_006088	1	1034107	T	C	N-syn	1	*FAM208B* [4]	c.4777A *>* G	p.K1593E	13	0	13	0	-	0
NC_006088	1	14729572	T	C	N-syn	0.8	*LAMB4*	c.4640A *>* G	p.K1547R	10	0	8	0	-	0
NC_006088	1	64181733	G	T	N-syn	1	*PIK3C2G*	c.1585G *>* T	p.A529S	10	0	0	-	10	0
NC_006088	1	65873551	C	G	N-syn	0.9	*LOC101748372*	c.960G *>* C	p.R320S	11	0	-	10	0	0
NC_006088	1	104629701	G	A	N-syn	1	*IFNAR2*	c.1099G *>* A	p.A367T	11	11	0	-	0	0
NC_006088	1	112303547	A	C	N-syn	0.8	*RPGR*	c.2209A *>* C	p.I737L	10	-	8	0	0	0
NC_006088	1	121349181	C	T|C	N-syn	0.75	*BMX*	c.[1549G *>* G]	p.D517N,	12	0	-	0	9	0
								+[1549G *>* A]	p.D517D						
NC_006088	1	127837266	G	T	N-syn	0.89	*ARSD*	c.449G *>* T	p.W150L	10	0	0	-	9	0
NC_006088	1	179358329	A	T|A	N-syn	0.76	*DDX10*	c.[1849T *>* T]	p.Y617N,	13	-	0	0	10	0
								+[1849T *>* A]	p.Y617Y						
NC_006088	1	185190031	T	C	N-syn	0.8	*CCDC67*	c.983A *>* G	p.Q328R	10	0	8	0	-	0
NC_006088	1	193338454	A	T	N-syn	1	*ART7B*	c.590T *>* A	p.L197Q	13	-	0	0	13	0
NC_006089	2	19385973	C	T	N-syn	1	*LOC420515*	c.3400G *>* A	p.V1134I	10	0	-	0	10	0
NC_006089	2	41279450	G	T	N-syn	1	*TOPAZ1*	c.2924C *>* A	p.T975N	10	0	0	-	10	0
NC_006089	2	42201510	T	T|C	N-syn	0.75	*PIK3R4*	c.[1937A *>* G]	p.D646D,	12	0	9	0	-	0
								+[1937A *>* A]	p.D646G						
NC_006089	2	63518627	T	C	N-syn	0.89	*LOC101751154*	c.137T *>* C	p.I46T	10	0	9	0	-	0
NC_006089	2	96924595	G	A	N-syn	1	*PTPN2*	c.934G *>* A	p.A312T	10	10	0	-	0	0
NC_006089	2	1.06E+08	G	C	N-syn	0.9	*COPN5L1*	c.17C *>* G	p.A6G	11	1	10	-	0	0
NC_006089	2	1.21E+08	T	C	N-syn	0.91	*LOC101751416*	c.194T *>* C	p.L65P	82	0	75	0	-	0
NC_006090	3	24479109	T	C	N-syn	1	*THADA*	c.859A *>* G	p.S287G	10	0	10	0	-	0
NC_006090	3	24515241	T	C	N-syn	1	*PLEKHH2*	c.1522T *>* C	p.F508L	10	0	10	0	-	0
NC_006090	3	37785831	A	G	N-syn	1	*TARBP1*	c.1321A *>* G	p.I441V	10	-	0	10	0	0
NC_006090	3	37785856	C	T	N-syn	1	*TARBP1*	c.1346C *>* T	p.T449I	10	0	-	0	10	0
NC_006090	3	38965704	G	T	N-syn	1	*DISC1*	c.1027C *>* A	p.L343I	10	0	0	-	10	0
NC_006090	3	105934067	C	G	N-syn	0.85	*GVINP1*	c.6146G *>* C	p.R2049T	14	0	-	12	0	0
NC_006091	4	120829955	C	T	N-syn	0.8	*LOC771752*	c.62G *>* A	p.R21Q	10	0	-	0	8	0
NC_006091	4	31100937	A	C	N-syn	0.9	*TTC29*	c.903T *>* G	p.D301E	11	-	10	0	0	0
NC_006091	4	56709491	G	G|A	N-syn	0.76	*C4H4ORF21*	c.[631G *>* G]	p.D211N,	13	10	0	-	0	0
								+[631G *>* A]	p.D211D						
NC_006091	4	60221486	C	T	N-syn	1	*BANK1*	c.934C *>* T	p.P312S	12	0	-	0	12	0
NC_006092	5	24297188	T	C	N-syn	1	*MGA*	c.7214T *>* C	p.M2405T	13	0	13	0	-	0
NC_006092	5	45169641	T	C	N-syn	1	*LOC100858625*	c.2191A *>* G	p.I731V	10	0	10	0	-	0
NC_006092	5	57915337	A	G	N-syn	0.86	*TMX1*	c.230A *>* G	p.D77G	15	-	0	13	0	0
NC_006093	6	130323	G	T	N-syn	1	*FAM13C*	c.1446G *>* T	p.E482D	10	0	0	-	10	0
NC_006093	6	23801282	T	C	N-syn	0.81	*ITPRIP*	c.1528A *>* G	p.I510V	11	0	9	0	-	0
NC_006094	7	4260582	C	T	N-syn	0.8	*ABCA12*	c.2110G *>* A	p.A704T	10	0	-	1	8	0
NC_006094	7	4717753	G	A	N-syn	0.8	*RAB17*	c.601G *>* A	p.V201I	10	8	0	-	0	0
NC_006094	7	4717765	G	A	N-syn	0.9	*RAB17*	c.613G *>* A	p.V205I	11	10	0	-	0	0
NC_006094	7	14344085	A	G	N-syn	1	*ZNF385B*	c.131A *>* G	p.H44R	13	-	0	13	0	0
NC_006094	7	15047308	A	G	N-syn	1	*LOC770919*	c.1253A *>* G	p.Y418C	10	-	0	10	0	0
NC_006094	7	15047322	A	G	N-syn	1	*LOC770919*	c.1267A *>* G	p.K423E	10	-	0	10	0	0
NC_006096	9	713606	G	A	N-syn	1	*SAG*	c.151G *>* A	p.V51M	13	13	0	-	0	0
NC_006096	9	20652272	T	C	N-syn	1	*SI*	c.2905T *>* C	p.S969P	11	0	11	0	-	0
NC_006096	9	21722478	G	A	N-syn	0.83	*LOC425015*	c.1352G *>* A	p.G451D	12	10	0	-	0	0
NC_006096	9	21722484	C	T	N-syn	0.8	*LOC425015*	c.1358C *>* T	p.P453L	10	0	-	0	8	0
NC_006096	9	22653344	T	G	N-syn	0.89	*DHX36*	c.333T *>* G	p.Y111.	10	0	0	9	-	1
NC_006097	10	11164703	C	T	N-syn	1	*FAM154B*	c.287G *>* A	p.R96K	11	0	-	0	11	0
NC_006098	11	9564860	G	A	N-syn	1	*LRP3*	c.2206G *>* A	p.G736R	10	10	0	-	0	0
NC_006099	12	5208897	A	G	N-syn	1	*LOC100857401*	c.3511A *>* G	p.K1171E	10	-	0	10	0	0
NC_006100	13	14403319	C	G	N-syn	0.9	*LOC101749661*	c.260G *>* C	p.G87A	11	0	-	10	0	0
NC_006101	14	1.2E+07	T	C	N-syn	1	*KIAA0556*	c.2794A *>* G	p.N932D	10	0	10	0	-	0
NC_006101	14	13770765	A	G	N-syn	0.89	*IFT140*	c.2372A *>* G	p.K791R	10	-	0	9	0	0
NC_006102	15	10822742	T	C	N-syn	0.89	*CCDC157*	c.1492A *>* G	p.S498G	10	1	9	0	-	0
NC_006104	17	6833124	C	T	N-syn	0.83	*GBGT1*	c.368G *>* A	p.R123H	12	0	-	0	10	0
NC_006104	17	7711131	A	G	N-syn	1	*PPP1R26*	c.637A *>* G	p.I213V	11	-	0	11	0	0
NC_006106	19	5656736	C	T	N-syn	0.8	*SLC46A1*	c.1174G *>* A	p.G392S	10	0	-	0	8	0
NC_006107	20	43121	C	T	N-syn	1	*IGSF1*	c.412C *>* T	p.R138C	10	0	-	0	10	0
NC_006107	20	478926	C	T	N-syn	0.8	*GGT7*	c.1472C *>* T	p.S491F	10	0	-	0	8	0
NC_006107	20	4706188	G	T	N-syn	0.89	*LOC101750167*	c.619G *>* T	p.A207S	10	0	0	-	9	0
NC_006108	21	1906952	T	C	N-syn	1	*TMEM52*	c.2T *>* C	p.M1T	10	0	10	0	-	0
NC_006108	21	1906991	G	T	N-syn	1	*TMEM52*	c.41G *>* T	p.C14F	11	0	0	-	11	0
NC_006108	21	2451062	T	G	N-syn	0.89	*SCNN1D*	c.1912A *>* C	p.I638L	10	0	0	9	-	0
NC_006108	21	3241081	C	T	N-syn	1	*SLC2A5*	c.1246G *>* A	p.A416T	11	0	-	0	11	0
NC_006108	21	4662973	C	A	N-syn	0.89	*EMC1*	c.1070G *>* T	p.S357I	10	9	-	0	0	0
NC_006111	24	130579	C	T|C	N-syn	0.81	*FEZ1*	c.[1196C *>* T]	p.P399P,	16	0	-	0	13	0
								+[1196C *>* C]	p.P399L						

Contig ID, chromosome (C) numbers, reference position (Ref Pos), reference base (Ref Base), called (SNP) base, impact (kinds of protein mutation), SNP%, feature name (gene name), DNA change, amino acid (AA) change, Depths, and five columns for SNP counts (Cnts) are indicated.

**Table 4:** Validation of SNPs using allele-specific PCR in 96 AR and 96 AP line chickens.

Chr	Ref Pos	Genes	Ref Base	Called Base	Impact	SNP%	Amino Acid Change	Results of 96 Birds Each from AP and AR Lines					
								Homozygous RJF		Heterozygote		Homozygous SNP	
								AR	AP	AR	AP	AR	AP
1	193338454	*ART7B*	A	T	N-syn	1	p.L197Q	5	93	23	3	68	0
1	121349181	*BMX*	C	T|C	N-syn	0.75	p.D517N, p.D517D	16	96	26	0	54	0
1	1034107	*FAM208B*	T	C	N-syn	1	p.K1593E	0	94	0	1	96	1
14	13770765	*IFT140*	A	G	N-syn	0.89	p.K791R	9	95	44	1	43	0
20	43121	*IGSF1*	C	T	N-syn	1	p.R138C	0	63	0	28	96	5
1	14729572	*LAMB4*	T	C	N-syn	0.8	p.K1547R	39	84	15	2	42	10
2	42201510	*PIK3R4*	T	T|C	N-syn	0.75	p.D646D, p.D646G	16	71	4	16	76	9
3	24479109	*THADA*	T	C	N-syn	1	p.S287G	0	64	0	30	96	2
2	41279450	*TOPAZ1*	G	T	N-syn	1	p.T975N	0	16	0	78	96	2

**Table 5:** Gene name and functions of genes containing amino acid changes showing over 10 depth counts in AR chickens.

ID	Entrez Gene Name	Location	Type(s)
*ABCA12*	ATP-binding cassette, sub-family A (ABC1), member 12	Plasma Membrane	Transporter
*ARSD*	arylsulfatase D	Cytoplasm	Enzyme
*BANK1*	B-cell scaffold protein with ankyrin repeats 1	Extracellular Space	Other
*BMX*	Bone Marrow on X chromosome (BMX) non-receptor tyrosine kinase	Cytoplasm	Kinase
*CCDC157*	Coiled-coil domain containing 157	Other	Other
*CCDC67*	coiled-coil domain containing 67	Other	Other
*DDX10*	DEAD (Asp-Glu-Ala-Asp) box polypeptide 10	Nucleus	Enzyme
*DHX36*	DEAH (Asp-Glu-Ala-His) box polypeptide 36	Cytoplasm	Enzyme
*DISC1*	disrupted in schizophrenia 1	Cytoplasm	Other
*EMC1*	Endoplasmic reticulum (ER) membrane protein complex subunit 1	Plasma Membrane	Other
*FAM13C*	Family with sequence similarity 13, member C	Other	Other
*FAM154B*	Family with sequence similarity 154, member B	Other	Other
*FAM208B*	Family with sequence similarity 208, member B	Other	Other
*FEZ1*	Fasciculation and elongation protein zeta 1 (zygin I)	Cytoplasm	Other
*GBGT1*	Globoside alpha-1,3-*N*-acetylgalactosaminyltransferase 1	Other	Enzyme
*GGT7*	Gamma-glutamyltransferase 7	Plasma Membrane	Enzyme
*GVINP1*	GTPase, very large interferon inducible pseudogene 1	Other	Other
*IFNAR2*	Interferon (α, β and Ω) receptor 2	Plasma Membrane	transmembrane receptor
*IFT140*	Intraflagellar transport 140	Extracellular Space	Other
*IGSF1*	Immunoglobulin superfamily, member 1	Plasma Membrane	Other
*ITPRIP*	Inositol 1,4,5-trisphosphate receptor interacting protein	Extracellular Space	Other
*KIAA0556*	KIAA0556	Extracellular Space	Other
*LAMB4*	Laminin, beta 4	Other	Other
*LRP3*	Low density lipoprotein receptor-related protein 3	Plasma Membrane	transmembrane receptor
*MGA*	MYC associated factor X (MAX) dimerization protein	Nucleus	transcription regulator
*PIK3C2G*	Phosphatidylinositol-4-phosphate 3-kinase, catalytic subunit type 2 γ	Cytoplasm	Kinase
*PIK3R4*	Phosphoinositide-3-kinase, regulatory subunit 4	Cytoplasm	Kinase
*PLEKHH2*	Pleckstrin homology domain containing, family H (with MyTH4 domain) member 2	Cytoplasm	Other
*PPP1R26*	Protein phosphatase 1, regulatory subunit 26	Nucleus	Other
*PTPN2*	Protein tyrosine phosphatase, non-receptor type 2	Cytoplasm	Phosphatase
*RAB17*	RAB17, member RAS oncogene family	Cytoplasm	Enzyme
*RPGR*	Retinitis pigmentosa GTPase regulator	Cytoplasm	Other
*SAG*	S-antigen; retina and pineal gland (arrestin)	Cytoplasm	Other
*SCNN1D*	Sodium channel, non-voltage-gated 1, δ subunit	Plasma Membrane	ion channel
*SI*	Sucrase-isomaltase (α-glucosidase)	Cytoplasm	Enzyme
*SLC2A5*	Solute carrier family 2 (facilitated glucose/fructose transporter), member 5	Plasma Membrane	Transporter
*SLC46A1*	Solute carrier family 46 (folate transporter), member 1	Plasma Membrane	Transporter
*TARBP1*	Trans-activation response (TAR) (HIV-1) RNA binding protein 1	Nucleus	transcription regulator
*THADA*	Thyroid adenoma associated	Other	Other
*TMEM52*	Transmembrane protein 52	Other	Other
*TMX1*	Thioredoxin-related transmembrane protein 1	Cytoplasm	Enzyme
*TOPAZ1*	Testis and ovary specific Piwi Argonaut and Zwille (PAZ) domain containing 1	Other	Other
*TTC29*	Tetratricopeptide repeat domain 29	Other	Other
*ZNF385B*	Zinc finger protein 385B	Nucleus	Other
*LOC101748372*	Uncharacterized	N/A	N/A
*ART7B*	Uncharacterized	N/A	N/A
*LOC420515*	Uncharacterized	N/A	N/A
*LOC101751154*	Uncharacterized	N/A	N/A
*COPN5L1*	Uncharacterized	N/A	N/A
*LOC101751416*	Uncharacterized	N/A	N/A
*LOC771752*	Uncharacterized	N/A	N/A
*C4H4ORF21*	Uncharacterized	N/A	N/A
*LOC100858625*	Uncharacterized	N/A	N/A
*LOC770919*	Uncharacterized	N/A	N/A

**Table 6:** Associated network functions of candidate genes.

ID	Molecules in Network	Score	Top Diseases and Functions
1	*ABCA12, ACP6, ARSB, ARSD, ARSE, ARSF, ARSG, ARSI, ARSJ, CBWD2, DDX10, DHX36, EMC1, GGT7, GNS, IFT122, IFT140, IFT172, ITPRIP, LAMB4, MGA, PANK2, PLEC, SCNN1D, SGSH, SLC46A1, SRPX, SUMF1, SUMF2, TARBP1, THADA,TTBK2*	30	Developmental Disorder, Hereditary Disorder, Metabolic Disease
	*TTC21B, UBC, WDR19*		
2	*ANO10, BANK1, BCR* (complex), *CAPN15, CCDC67, CCDC141, CCDC85B, DISC1, FAM208B, FAS, FEZ1, GPR107, IGSF1, KMT2D, LYN, MARVELD1, NT5DC1, OARD1, P2RY8, PLEKHH2, PPP1R26*	22	Cell Death and Survival, Hematological System Development and Function, Humoral Immune Response
	*RELL1, RPGR, SLC13A3, SLC26A11, SLC35F5, SMC3, SYPL2, TGFB1, MEM184B, TMX1, TRMT2B, UBC, ZNF761, ZNF385B*		
3	*ARRB1, BMX, Cgm4/Psg16, CRK-CRKL-CBL, Erbb3* dimer, *FAM13C, FRY, GAB1/2, GHR dimer, GSN-PI3K-PIP2-Src, Hcst dimer, IFNAR2, Insulin, IRS2-PI3K, LMNA, LRP, LRP3, LRP10, Met dimer, NFkB* (complex), *NUPR1, PI3K* (complex), *PIK3C2G, PIK3R4, Prl4a1, PTPN2, RAB17, SAG, Sh2b3, SHC-GRB2-GAB1, SHP2-PI3K-GAB2, SI, SLC2A5, T3-TRβ, Vegfr* dimer	22	Developmental Disorder, Gastrointestinal Disease, Hereditary Disorder

## Supplementary Materials

The following are available online at http://www.mdpi.com/2073-4425/9/10/512/s1, Table S1: Functional groups of genes containing SNPs uniquely found in AR chickens.

## References

[1] Andersson L. (2001). Genetic dissection of phenotypic diversity in farm animals. Nat. Rev. Genet..

[2] Andersson L., Georges M. (2004). Domestic-animal genomics: deciphering the genetics of complex traits. Nat. Rev. Genet..

[3] Fan W., Ng C.S., Chen C., Lu M.J., Chen Y., Liu C., Wu S., Chen C., Chen J., Mao C. (2013). Genome-wide patterns of genetic variation in two domestic chickens. Genome Biol. Evol..

[4] Li D., Lian L., Qu L., Chen Y., Liu W., Chen S., Zheng J., Xu G., Yang N. (2013). A genome-wide SNP scan reveals two loci associated with the chicken resistance to Marek’s disease. Anim. Genet..

[5] Zhou H., Mitchell A., McMurtry J., Ashwell C., Lamont S.J. (2005). Insulin-like growth factor-I gene polymorphism associations with growth, body composition, skeleton integrity, and metabolic traits in chickens. Poult. Sci..

[6] Kong H.R., Anthony N.B., Rowland K.C., Khatri B., Kong B.C. (2018). Genome re-sequencing to identify single nucleotide polymorphism markers for muscle color traits in broiler chickens. Asian-Australas. J. Anim. Sci..

[7] Ou J., Tang S., Sun D., Zhang Y. (2009). Polymorphisms of three neuroendocrine-correlated genes associated with growth and reproductive traits in the chicken. Poult. Sci..

[8] Dorshorst B., Molin A., Rubin C., Johansson A.M., Strömstedt L., Pham M., Chen C., Hallböök F., Ashwell C., Andersson L. (2011). A complex genomic rearrangement involving the endothelin 3 locus causes dermal hyperpigmentation in the chicken. PLoS Genet..

[9] Wright D., Boije H., Meadows J.R., Bed’Hom B., Gourichon D., Vieaud A., Tixier-Boichard M., Rubin C., Imsland F., Hallböök F. (2009). Copy number variation in intron 1 of *SOX5* causes the pea-comb phenotype in chickens. PLoS Genet..

[10] Hayden A. (2016). Identification of biomarkers associated with Rous sarcoma virus-induced tumors in two divergently selected chicken lines. Master’s Thesis.

[11] Devaney J.A., Gyles N.R., Lancaster J.L. (1982). Evaluation of Arkansas Rous sarcoma regressor and progressor lines and giant jungle fowl for genetic resistance to the northern fowl mite. Poult. Sci..

[12] Sun W., Yang J. (2010). Functional mechanisms for human tumor suppressors. J. Cancer.

[13] Taylor R.L. (2004). Major histocompatibility (B) complex control of responses against Rous sarcomas. Poult. Sci..

[14] Rubin C., Zody M.C., Eriksson J., Meadows J.R., Sherwood E., Webster M.T., Jiang L., Ingman M., Sharpe T., Ka S. (2010). Whole-genome resequencing reveals loci under selection during chicken domestication. Nature.

[15] Sachidanandam R., Weissman D., Schmidt S.C., Kakol J.M., Stein L.D., Marth G., Sherry S., Mullikin J.C., Mortimore B.J., Willey D.L. (2001). A Map of human genome sequence variation containing 1.42 million single nucleotide polymorphisms. Nature.

[16] Shastry B.S. (2009). SNPs: Impact on gene function and phenotype. Single Nucleotide Polymorphisms. Methods Mol. Biol..

[17] Syvanen A.C. (2001). Accessing genetic variation: genotyping single nucleotide polymorphisms. Nat. Rev. Genet..

[18] Malek M., Hasenstein J., Lamont S. (2004). Analysis of chicken *TLR4, CD28, MIF*, *MD-2,* and *LITAF* genes in a salmonella enteritidis resource population. Poult. Sci..

[19] Zhang L., Li P., Liu R., Zheng M., Sun Y., Wu D., Hu Y., Wen J., Zhao G. (2015). The identification of loci for immune traits in chickens using a genome-wide association study. PLoS ONE.

[20] Luo C., Qu H., Ma J., Wang J., Hu X., Li N., Shu D. (2014). A genome-wide association study identifies major loci affecting the immune response against infectious bronchitis virus in chicken. Infect. Genet. Evol..

[21] Andrews S. FastQC: A Quality Control Tool for High Throughput Sequence Data. Reference Source. http://www.bioinformatics.babraham.ac.uk/projects/fastqc/.

[22] Bushnell B. BBMap Short Read Aligner. http://sourceforge.net/projects/bbmap.

[23] Jang H.M., Erf G.F., Rowland K.C., Kong B.W. (2014). Genome resequencing and bioinformatic analysis of SNP containing candidate genes in the autoimmune vitiligo Smyth line chicken model. BMC Genom..

[24] Liu J., Huang S., Sun M., Liu S., Liu Y., Wang W., Zhang X., Wang H., Hua W. (2012). An improved allele-specific PCR primer design method for SNP marker analysis and its application. Plant Methods.

[25] Kong B.W., Lee J.Y., Bottje W.G., Lassiter K., Lee J., Foster D.N. (2011). Genome-wide differential gene expression in immortalized DF-1 chicken embryo fibroblast cell line. BMC Genom..

[26] Pértille F., Guerrero-Bosagna C., Da Silva V.H., Boschiero C., da Silva Nunes J.D.R., Ledur M.C., Jensen P., Coutinho L.L. (2016). High-throughput and cost-effective chicken genotyping using next-generation sequencing. Sci. Rep..

[27] Popovic D., Vucic D., Dikic I. (2014). Ubiquitination in disease pathogenesis and treatment. Nat. Med..

[28] Courtney K.D., Corcoran R.B., Engelman J.A. (2010). The PI3K pathway as drug target in human cancer. J. Clin. Oncol..

[29] Hennessy B.T., Smith D.L., Ram P.T., Lu Y., Mills G.B. (2005). Exploiting the PI3K/AKT pathway for cancer drug discovery. Nat. Rev. Drug Discov..

[30] Liu P., Cheng H., Roberts T.M., Zhao J.J. (2009). Targeting the phosphoinositide 3-Kinase pathway in cancer. Nat. Rev. Drug Discov..

[31] Wee S., Jagani Z., Xiang K.X., Loo A., Dorsch M., Yao Y.M., Sellers W.R., Lengauer C., Stegmeier F. (2009). PI3K pathway activation mediates resistance to MEK inhibitors in KRAS mutant cancers. Cancer Res..

[32] Ismail H.A., Lessard L., Mes-Masson A.M., Saad F. (2004). Expression of NF-kappaB in prostate cancer lymph node metastases. Prostate.

[33] Dolcet X., Llobet D., Pallares J., Matias-Guiu X. (2005). NF-kB in development and progression of human cancer. Virchows Arch..

[34] Levine A.J., Finlay C.A., Hinds P.W. (2004). P53 is a tumor suppressor gene. Cell.

[35] Grandage V.L., Gale R.E., Linch D.C., Khwaja A. (2005). PI3-kinase/Akt is constitutively active in primary acute myeloid leukaemia cells and regulates survival and chemoresistance via NF-kappaB, Mapkinase and p53 Pathways. Leukemia.

[36] Potter D.S., Kelly P., Denneny O., Juvin V., Stephens L.R., Dive C., Morrow C.J. (2014). BMX acts downstream of PI3K to promote colorectal cancer cell survival and pathway inhibition sensitizes to the BH3 mimetic ABT-737. Neoplasia.

[37] Qiu Y., Kung H.J. (2000). Signaling network of the Btk family kinases. Oncogene.

[38] Vogt P.K., Hart J.R. (2011). PI3K and STAT3: A new alliance. Cancer Discov..

